# Evaluation of Neuro-Hormonal Dynamics after the Administration of Probiotic Microbial Strains in a Murine Model of Hyperthyroidism

**DOI:** 10.3390/nu16071077

**Published:** 2024-04-06

**Authors:** Sorina Nicoleta Voicu, Anca Ioana (Amzăr) Scărlătescu, Miruna-Maria Apetroaei, Marina Ionela (Ilie) Nedea, Ionuț Emilian Blejan, Denisa Ioana Udeanu, Bruno Ștefan Velescu, Manuela Ghica, Octavian Alexandru Nedea, Călin Pavel Cobelschi, Andreea Letiția Arsene

**Affiliations:** 1 Department of Biochemistry and Molecular Biology, Faculty of Biology, University of Bucharest, Splaiul Independenței 91–95, 050095 Bucharest, Romania; sorina.voicu@bio.unibuc.ro; 2 Faculty of Pharmacy, “Carol Davila” University of Medicine and Pharmacy, 6 Traian Vuia Street, 020956 Bucharest, Romania; anca-ioana.amzar@drd.umfcd.ro (A.I.S.); marina.nedea@umfcd.ro (M.I.N.); denisa.udeanu@umfcd.ro (D.I.U.); bruno.velescu@umfcd.ro (B.Ș.V.); manuela.ghica@umfcd.ro (M.G.); andreea.arsene@umfcd.ro (A.L.A.); 3 Faculty of Biotechnical Systems Engineering, University Politehnica of Bucharest, Splaiul Independentei 313, 060042 Bucharest, Romania; octavian.nedea@stud.isb.upb.ro; 4 Faculty of Medicine, Transilvania University, Bulevardul Eroilor 29, 500036 Brașov, Romania

**Keywords:** probiotics, microbiota, hyperthyroidism, neurotransmitters, murine model

## Abstract

The microbiota–gut–brain axis has received increasing attention in recent years through its bidirectional communication system, governed by the ability of gut microorganisms to generate and regulate a wide range of neurotransmitters in the host body. In this research, we delve into the intricate area of microbial endocrinology by exploring the dynamic oscillations in neurotransmitter levels within plasma and brain samples. Our experimental model involved inducing hyperthyroidism in mice after a “probiotic load” timeframe using two strains of probiotics (*Lactobacillus acidophilus*, *Saccharomyces boulardii*, and their combination). These probiotic interventions continued throughout the experiment and were intended to uncover potential modulatory effects on neurotransmitter levels and discern if certain probiotic strains exhibit any protection from hyperthyroidism. Moreover, we aimed to outline the eventual connections between the gut microbiota and the hypothalamus–pituitary–thyroid axis. As our study reveals, there are significant fluctuations in crucial neurotransmitters within the hyperthyroidism model, related to the specific probiotic strain or combination. These findings could support future therapeutic approaches, help healthcare professionals choose between different probiotic therapies, and also allow us proceed with caution when administering such treatments, depending on the health status of hyperthyroid patients.

## 1. Introduction

The gastrointestinal tract houses the biggest virtual organ in the body, known as the intestinal microbiota. The microbiome, which refers to the combined genetic material of bacteria, is 100 times larger than the human genome [1,2]. Bacteria are the predominant microorganisms found in the gut, and they coexist with viruses, fungi, and protozoa to create the gut microbiota [3,4].

The gut microbiota is involved in host processes like digestion, xenobiotic metabolism, maintenance of the integrity of the intestinal mucosal barrier, immune regulation, and defence mechanisms against pathogens [5,6].

Thyroid disorders are common within the general population and also subject to the treatment non-adherence of patients, since thyroid disease is often accompanied by comorbidities like hypertension and diabetes, making it difficult for patients to stick to their medication [7]. Autoimmune thyroid diseases (ATDs) are some of the most common organ-specific, frequently encountered autoimmune disorders, with an increasing prevalence of more than 5% worldwide [8]. Several studies have shed light on the crucial role of an altered microbiota in the onset of many different autoimmune conditions, suggesting its involvement in pathogenesis [9,10,11].

The gut microbiota may influence the hypothalamus–pituitary axis into releasing TSH, thus playing a role in thyroid diseases. Overgrowth or imbalance of the gut microbiota was found to be associated with hypothyroid Hashimoto’s thyroiditis patients instead of euthyroid patients. The persistent accumulation of metabolites, which appeared because of gut dysbiosis, is presumed to be an important cause of thyroid dysfunction [12].

Probiotic supplementation has shown beneficial effects on thyroid hormones and overall thyroid function [13]. In hypothyroidism and hyperthyroidism, the number of bacteria from the genera *Lactobacillaceae* and *Bifidobacteriaceae* is generally decreased at the gut level. Supplementation with *Lactobacillus reuteri* has been shown to improve thyroid function in mice by increasing free T4 (total thyroxine), thyroid mass, and physiological parameters, such as more active behaviour [14]. Imbalances in gut bacteria are frequently observed in individuals with thyroid disorders. It first modifies the immune response by promoting inflammation and decreasing immune tolerance, resulting in harm to the intestinal membrane and an increase in intestinal permeability. This, in turn, not only leads to heightened exposure to antigens but also triggers local inflammation. Additionally, it has the potential to impact thyroid hormone levels by means of its deiodinase activity and by inhibiting TSH [15].

The microbiota plays a significant role in various aspects of thyroid function, including iodine uptake, the enterohepatic cycling of thyroid hormones, and the impact on thyroid-targeted medication. It can affect the bioavailability of L-thyroxine, which is commonly used to treat hypothyroid patients, as well as the efficacy and toxicity of the antihyperthyroid drug propylthiouracil [16].

Since a large part of the immune system’s response to environmental factors is mediated by bacterial ecosystems, a healthy gut microbiota can have a profound effect on the body’s immunity, influenced by neurotransmitters such as GABA, catecholamines, and serotonin [17,18,19]. Our approach focused on the study of the microbiome defines disease as a loss of commensal microbes involved in maintaining general health and the excessive multiplication of pathogenic bacteria. These negative changes in bacterial populations can explain an increased susceptibility to thyroid imbalances, diabetes, cancer, sepsis, and depression [17,20]. Regarding the gut–brain and gut–thyroid axes, a topic of interest in the literature is how the microbiota is capable of producing, secreting, and recognising neuroactive compounds with similar structures to those released by the host, including dopamine, adrenaline, noradrenaline, serotonin, and GABA [21,22]. The modulation of neurotransmitter levels by bacteria can impact host physiology, and preliminary studies in humans show that interventions in microbiota structure can also alter neurotransmitter levels [23]. The interaction between the microbiome and the neurophysiological network via hormonal signals is commonly referred to as microbial endocrinology or interkingdom communication. This is the main mechanism by which the microbiota and the host impact one another [24].

About 90% of hyperthyroid disorders have an autoimmune component, likely triggered by microbiome dysbiosis. Furthermore, hyperthyroidism (HT) has been linked to decreases in microbial populations of *Bifidobacterium* spp. and *Lactobacillus* spp., but with expansions of *Enterococcus* spp. populations, likely through thyroid-reactive antigenic molecular mimicry [25]. It is recognised that the administration of antibiotics can cause dysbiosis, contributing to the loss of resistance to colonisation, followed by an increase in resistance in the intestinal microbiota. The entire range of antibiotic resistance genes (resistomes) in a specific microbiota are found in both pathogenic and non-pathogenic bacteria [26]. Regardless of the cause, dysbiosis opens a vicious circle of perpetuation of antibiotic resistance determinants and variation in intestinal membrane permeability in the case of inflammatory bowel diseases, which can be accompanied by disturbances to the hypothalamic–pituitary–thyroid (HPT) axis [27].

The objective of this study was to systematically evaluate the neurohormonal dynamics in an experimental model of hyperthyroidism, particularly focusing on the effects following the administration of specific probiotic microbial strains.

## 2. Materials and Methods

### 2.1. Animal Selection and Housing Conditions

All the procedures and protocols carried out during the experiment followed Directive 2010/63/EU and Romanian Law 43/2014 regarding the use of animals for scientific purposes. This research was also conducted in accordance with ARRIVE guidelines, and all experimental procedures and protocols received approval from the Ethical Committee for Scientific Research of the “Carol Davila” University of Medicine and Pharmacy Bucharest, Romania (approval no. 38032/9 December 2022).

For this investigation, a group of 60 adult mice from the NMRI strain that were 90 days old were chosen as the experimental subjects. These mice had a body weight of 23 ± 3 grammes and were sourced from the biobase at the “Cantacuzino” National Institute of Medical and Military Research and Development.

To ensure these conditions, all animal-related procedures were conducted in a controlled laboratory environment. The temperature and humidity levels in this environment were consistently monitored using a thermohygrometer. The temperature was maintained between 20 °C and 22 °C, while humidity was regulated between 35% and 45%. This approach aims to minimise any effects resulting from fluctuations in temperature or humidity.

Moreover, following established practices in animal research, a three-day acclimatisation period was provided for the rodents. During the period of adjustment, they were provided access to food and water ad libitum. This three-day acclimatisation period is a well-established practice used in order to minimise any stress-related impact on the accuracy of our findings [28,29]. 

### 2.2. Tested Compounds and Dosages

The study involved the administration of various compounds, including probiotic bacteria (*Lactobacillus acidophilus*), probiotic yeasts (*Saccharomyces boulardii*), combinations of probiotics (*Lactobacillus acidophilus* and *Saccharomyces boulardii*), thiamazole (methimazole), and sodium levothyroxine. The doses were administered as follows: 100 µL of bacterial suspension containing 10^7^ CFU/day *per os* (p.o.) [30,31], 2 μg/mL of levothyroxine in drinking water [32,33], and 0.04% (*w*/*v*) thiamazole (methimazole) in drinking water [34,35]. The chosen probiotic dosage of 10^7^ CFU is based on scientific precedent, balancing efficacy and safety to modulate the gut microbiota and impact autoimmune and endocrine disorders effectively without causing any adverse effects.

To facilitate result quantification and comparative analysis, two reference groups were included in the study. One group received thiamazole, a medication commonly used in hyperthyroidism treatment, while the other group served as a control and received a physiological saline solution.

### 2.3. Experimental Design and Group Allocation

After the acclimatisation period, the animals were split into six groups, with each group containing 10 mice, housed together in two cages, with 5 mice per cage. The animals received their treatment in accordance with their assigned experimental groups. Throughout the experiment, their body weight was closely monitored as an indicator of their well-being and response to the treatment, and every effort was made to minimise the stress and suffering linked to oral administration. The study consisted of two phases, a pre-treatment period lasting 16 days, followed by a treatment period of 14 days, resulting in an overall experimental period of 30 days. The initial 16-day pre-treatment phase also served as the ‘probiotic loading’ period. This timeframe was selected based on multiple research studies indicating that, when administered consistently over a span of days to weeks, probiotics gradually influence the composition and variety, and finally regulate the functionality of the gut microbiota [36,37,38,39].

Group 1 (M, healthy control/absolute control) received a physiological saline solution in an amount of 0.1 mL/10 g of body weight for 30 days. Group 2 (E, hyperthyroidism control) and Group 6 (EThy, treated hyperthyroidism) received the same treatment as group M for the first 16 days. Groups 3 to 5 (ELb, ESch, and ELbSch) received 100 µL of probiotic suspension containing 10^7^ CFU/day as follows: Group 3 (ELb)—*Lactobacillus acidophilus* suspension; Group 4 (Esch)—*Saccharomyces boulardii* suspension; and Group 5 (ELbSch)— combined therapy with a *Lactobacillus acidophilus* and *Saccharomyces boulardii* suspension.

On the 17th day of the experimental period, rodents belonging to groups 2–6 underwent oral sodium levothyroxine exposure, a well-documented inducer of hyperthyroidism [32]. Specimens belonging to Group 6 also received concomitant therapy with thiamazole. By administering thiamazole simultaneously with the induction of hyperthyroidism, we aimed to create a comparative scenario where the effects of probiotics could be cross-evaluated against a hyperthyroidism standard treatment. This supported our study in allowing an extensive assessment of whether or not probiotics could act as a preventive factor for hyperthyroidism disease and also as an alternative or comparator agent in managing already established hyperthyroidism.

Treatment was stopped 24 h before sacrifice. Upon completion of the treatment regimen, the animals were euthanized through decapitation to collect tissue samples for further analysis. Specifically, the brain and blood were sampled to assess the extent of protection conferred by the probiotic interventions. The blood samples were left for 30 min to settle, then centrifuged at 3500 rpm for 5 min to separate plasma. Finally, the plasma was collected for further analysis. The sampled organs and plasma were stored at −80 °C in order to be analysed using ELISA kits for the detection and dosage of the following hormones and neurotransmitters: adrenocorticotropic hormone (ACTH), cortisol, epinephrine/adrenaline (N), noradrenaline/norepinephrine (NA), serotonin (5HT), dopamine (DA), histamine (Hys), and gamma-aminobutyric acid (GABA).

ACTH levels were used to confirm the feasibility of the chosen hyperthyroidism model, since low levels of this hormone lead to an inhibition of the HPA axis and cause the consecutive activation of the HPT axis [40,41], manifested by stimulation of the thyroxine-5′-deiodinase type II enzyme activity [42]. Thyroxine-5′-deiodinase is a type of enzyme that converts the prohormone thyroxine (T4) into active triiodothyronine (T3) [43].

### 2.4. Monitoring of Physiological Parameters

A systematic observation of body weight was conducted in order to assess the physical health effects of inducing hyperthyroidism and probiotic administration on the experimental subjects. Prior to each weighing session, a calibrated precision electronic scale was utilised to determine the weights with accuracy. At the beginning of the study (on day one) and at predetermined intervals thereafter, the weight of each rodent was recorded for the duration of the experiment: upon the introduction of the probiotic load, upon the induction of hyperthyroidism, and throughout the treatment phase. In order to reduce stress and its potential influence on weight, the mice were gently restrained and positioned on the sharp scale, which was enclosed by Plexiglas panels, during each weighing session. Weight information was documented in grammes with a precision of 0.1 g.

Across all weighing days, data were analysed as the mean per group in order to identify significant changes and trends in body weight across all groups.

Based on data in the scientific literature, a new well-being scale was created to evaluate the animals’ overall well-being and level of activity. This measure was intended to assess a wide variety of physiological and behavioural indicators related to stress, well-being, and activity level. The significance of behavioural expression as an indicator of animal welfare is elaborated by Boissy et al. [44] and Mellor and Beausoleil [45], who support the development of this scale by emphasising the importance of evaluating both positive and negative states in the assessment of animal welfare. Furthermore, the use of physiological indicators is consistent with Sherwin’s recommendations, which support the incorporation of physiological data into assessments of animal welfare in order to better comprehend the impacts of experimental models [46].

Each individual was observed in person by two impartial observers in order to determine their welfare. To avoid bias, observers were blinded to the treatment groups. Prior to the study, observers went through a calibration session where they discussed standard behaviours and decided on scoring standards. To establish a relationship between changes in physical health and improvements in behavioural and physiological well-being, observations were conducted concurrently with body weight assessments.

A range of factors, including fur and eye health, activity, water and food consumption, and aggressiveness, were assessed using the scoring system. The scoring of each component was conducted using a scale that was intended to measure the intensity or frequency of observed behaviours. The scores, analysed parameters, and averages for the interpretation of the results are presented in Table 1.

Each observer’s scores were recorded separately and then averaged for analysis, resulting in a data set for assessing the effects of probiotic therapy and hyperthyroidism induction on animal welfare.

### 2.5. Hormones and Neurotransmitters Analysis

The gut microbiota is involved in the synthesis and release regulation of neurotransmitters via mechanisms such as bacterial production or the conversion of specific substrates, or via acting as signalling molecules [23,47]. The primary objective was to investigate the effects of probiotics on neurotransmitters in a murine model of hyperthyroidism. Specifically, we aimed to examine how probiotics modulate the balance of the gut microbiota and determine whether or not probiotics have a protective effect against neurotransmitter-level abnormalities commonly observed in individuals with hyperthyroidism. The objective of this study was to assess the associations between the central and peripheral dynamics of neuroactive substances following probiotic delivery by measuring neurotransmitters in both the brain and plasma.

These hormone and neurotransmitter analyses were performed in accordance with the manufacturer’s instructions on competitive enzyme-linked immunosorbent assay kits as follows: mouse adrenocorticotropin hormone (ACTH) (Abbexa Ltd., Cambridge, UK, reference range: 2.35–1000 pg/mL), cortisol (Abbexa Ltd., Cambridge, UK, reference range: 12.5–800 ng/mL), epinephrine/adrenaline (N) (Abbexa Ltd., Cambridge, UK, reference range: 24.69–2000 pg/mL), noradrenaline/norepinephrine (NA) (Abbexa Ltd., Cambridge, UK, reference range: 15.6–1000 pg/mL), serotonin (5HT) (Abbnova, GmbH, Taipei, Taiwan, reference range: 10.2–2500 ng/mL), dopamine (D) (Abbexa Ltd., Cambridge, UK, reference range: 0.156–10 ng/mL), histamine (Hys) (Wuhan Fine Biotech Co., Ltd., Hubei, China, reference range: 1.563–100 ng/mL), gamma-aminobutyric acid (GABA) (Wuhan Fine Biotech Co., Ltd., Hubei, China, reference range: 31.25–2000 pg/mL).

The absorbance was read at 450 nm using FlexStation 3 Multi-Mode Microplate Reader from Molecular Devices LLC and the SoftMax Pro software (version 7.1.2).

### 2.6. Statistical Analysis

Statistical analysis and graphs were implemented using the R statistical software (R version 4.1.3) [48]. The statistical methods one-way ANOVA or the Kruskal–Wallis test were used in the case of continuous dependent variables in relation to the independent variable group. For the small data (10 values per lot), we checked the conditions of normality with the Shapiro–Wilk test and the conditions related to heteroscedasticity with the Levene Test. The multiple-comparisons method for the post hoc analysis was used to highlight the differences between the experimental group and the rest of the groups with Tukey-type tests.

We highlighted the influence of the independent variable time or group on the dependent continuous variables with significant *p* values attached in the illustrated boxplot-type graphs and the interaction plot-type graphs. We generated a spider-type chart to further analyse the behaviour of the dependent qualitative variables (fur, aggressiveness, feeding, activity, and eyes) in relation to the independent variables group and time (first and second periods).

The results were considered significant if the obtained *p* values were below the statistical level of 5%.

In order to assess fluctuations in hormone and neurotransmitter levels, we quantified them as percentages (%), comparing these with those of both the control group (E) and the EThy group.

## 3. Results

### 3.1. Monitoring of Physiological Parameters

**Figure 1:**
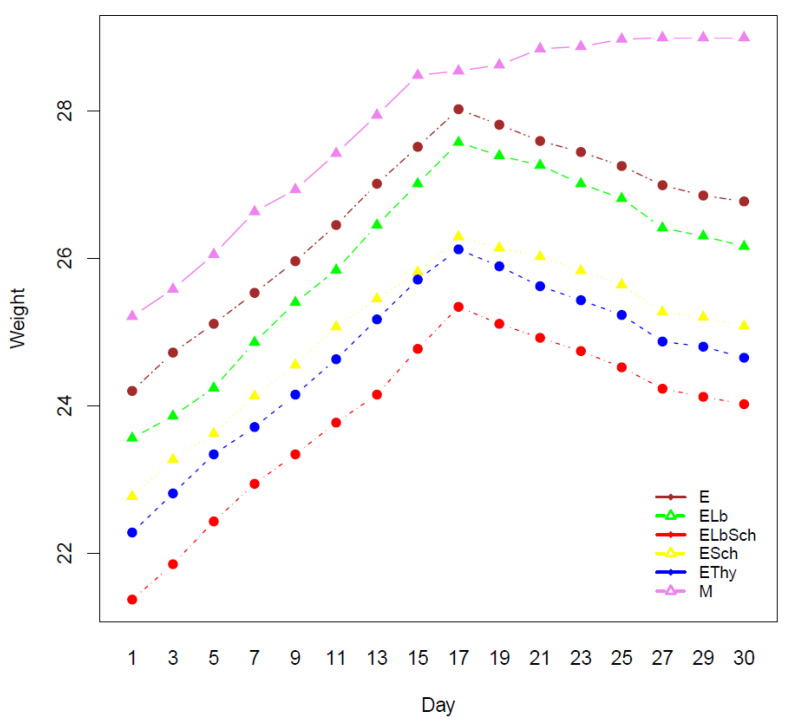
Interaction plot of weight incidence.

The weight fluctuations observed throughout the study period underscored the effect of hyperthyroidism on the body (Figure 1). Initial weight gain in all groups, including those subsequently diagnosed with hyperthyroidism, was indicative of development and good health. This pattern persisted until day 17, when hyperthyroidism was introduced. An important observation was the weight loss that occurred within the hyperthyroidism groups at this time. On the contrary, the healthy control group exhibited sustained weight gain in the absence of hyperthyroidism.

**Figure 2:**
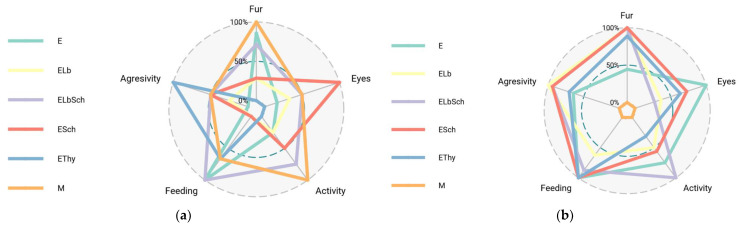
Comparative welfare assessment before and after hyperthyroidism induction. (**a**) Welfare assessment days 1–16. (**b**) Welfare assessment days 17–30.

Figure 2 depicts the comparative welfare assessment before and after hyperthyroidism induction.

Prior to the induction of hyperthyroidism, the radar map displayed scores that varied throughout all categories. The control group, denoted as M, exhibited elevated scores across all domains, suggesting a state of excellent health and absence of discomfort. The observed patterns in the experimental groups (E, ELb, ELbSch, ESch, and EThy) suggest that the probiotics did not induce any significant changes in the measured well-being indices.

Following the initiation of hyperthyroidism, the radar map exhibits distinct variations among the several groups. The experimental group exhibiting hyperthyroidism (E) demonstrates a decrease in physical activity levels and fur condition scores, indicating the impact of stress and physiological alterations associated with hyperthyroidism. In contrast, the experimental groups administered with probiotics (ELb, ELbSch, and ESch) exhibited scores in these domains, suggesting that probiotic therapy potentially mitigated certain adverse consequences associated with hyperthyroidism. The EThy group, which underwent hyperthyroidism therapy, had scores indicating a degree of improvement.

### 3.2. Variation in Dosed Parameters

Table 2 systematises the concentrations of the dosed parameters as mean ± standard deviation (SD).

**Figure 3:**
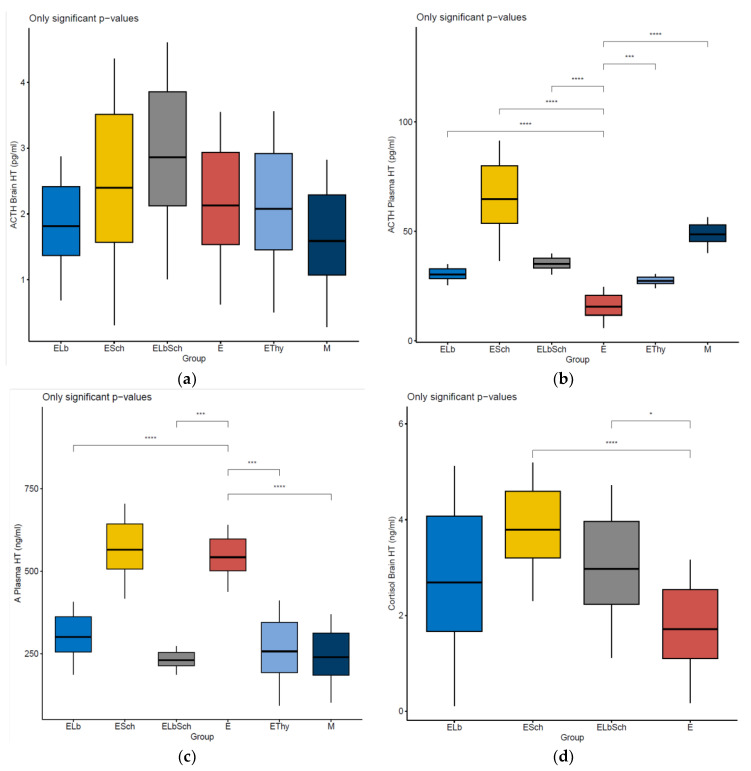

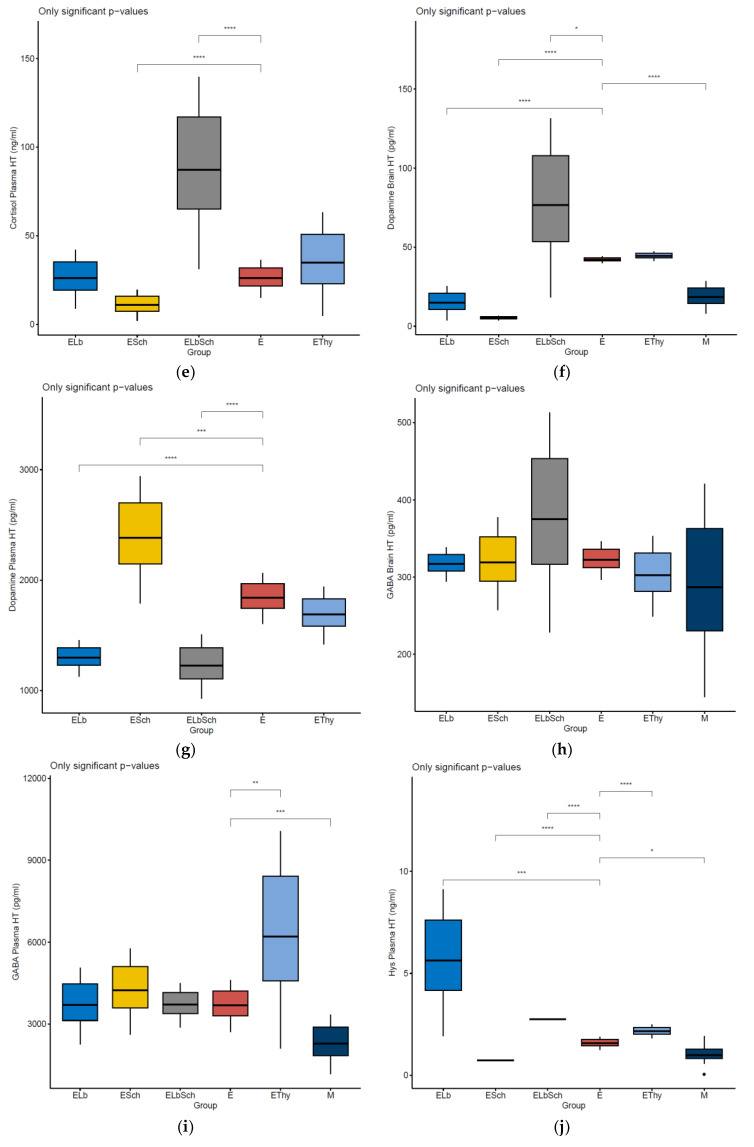

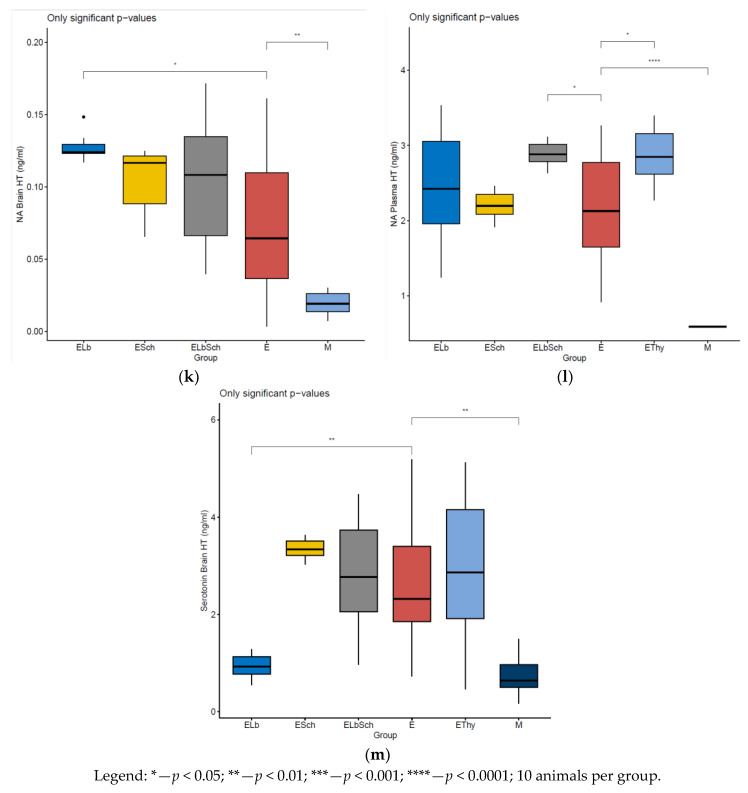
Mean values of concentrations of dosed markers in plasma and brain samples from experimental groups of the murine model of hyperthyroidism. (**a**) ACTH Brain HT. (**b**) ACTH Plasma HT. (**c**) A Plasma HT. (**d**) Cortisol Brain HT. (**e**) Cortisol Plasma HT. (**f**) Dopamine Brain HT. (**g**) Dopamine Plasma HT. (**h**) GABA Brain HT. (**i**) GABA Plasma HT. (**j**) Hys Plasma HT. (**k**) NA Brain HT. (**l**) NA Plasma HT. (**m**) Serotonin Brain HT.

There were no observed variations in the concentrations of ACTH in the brain across the experimental groups during analysis. The findings indicate that the treatments had no significant effect on ACTH levels in brain tissue, as shown in Figure 3a.

When examining the ACTH concentrations in plasma, notable variations were observed between the E group and the multiple treatment groups. There was a highly significant change in ACTH levels observed in the M, ELb, Esch, and ELbEsch groups (*p* < 0.0001), and a pronounced difference was seen in the EThy group (*p* < 0.001), as shown in Figure 3c.

There were variations in the levels of adrenaline in the plasma when comparing the E group with the other experimental groups. The M and ELb groups exhibited a statistically significant decrease in levels (*p* < 0.001). Significant decreases were observed when comparing group E with EThy and ELbSch (*p* < 0.01), as shown in Figure 3c.

Further analysis focusing on cortisol levels both in plasma and brain tissues revealed a few variations across all study groups, as shown in Figure 3d,e. In matters of brain cortisol, there was a highly significant increase when comparing E with Esch (*p* < 0.0001). On the other hand, in plasma cortisol levels, there was a significant statistical difference when comparing E with ESch and ELbSch (*p* < 0.0001). These findings indicate that the treatments did not have an important and quantifiable impact on concentrations and maintained consistency within expected physiological ranges.

Upon analysing dopamine levels in brain tissues, notable variations were observed between the ELb and ESch groups and the E group. These experimental groups showed a highly significant change in brain dopamine concentrations (*p* < 0.0001), as shown in Figure 3f. It can be inferred that the ESch treatment might affect dopamine regulation in the brain. We observed variations in plasma dopamine levels between E and the groups treated with *Lactobacillus* (ELb) and the combination of *Lactobacillus acidophilus* and *Saccharomyces boulardii* (ELbSch). Both treated groups exhibited alterations in dopamine levels, with statistical significance (*p* < 0.0001 for each comparison). The observed variations, depicted in Figure 3g, indicate the potential impact of the treatment on the regulation of systemic dopamine.

There were no significant differences in brain tissue GABA concentrations observed among all groups. The data in Figure 3h suggest that the various treatments had a limited impact on the levels in the brain. The consistent concentrations observed offer strong evidence for the impact of these treatments on the inhibitory neurotransmitter within the central nervous system, thus providing support for our conclusion.

The analysis of plasma GABA levels revealed consistent profiles across all groups, without any significant variations observed in the probiotic-treated groups. The data indicate that the treatments had no effect on plasma GABA levels, as depicted in Figure 3i.

Our research reveals notable variations in histamine plasma levels among the groups examined. Highly significant increases were observed in group E when compared with the ELb, ELbSch, and EThy groups (*p* < 0.0001). There was an extremely notable decrease observed in comparison with the ESch group (*p* < 0.0001). These results suggest that plasma histamine regulatory mechanisms were substantially affected by the treatments, as presented in Figure 3j.

Consistent levels of concentrations within noradrenaline brain tissues were observed across all treatment groups, indicating no significant changes, except from ELb (*p* < 0.05), as presented in Figure 3k. When analysing plasma levels, no significant changes were found among the experimental groups, except from ELbSch (*p* < 0.5). These findings suggest that the treatments had a minimal impact on plasma noradrenaline levels. The levels across all groups are illustrated in Figure 3l, showcasing the findings.

There were notable differences in serotonin levels when comparing E with both the M group and the ELb group. There were observed variations in serotonin levels between the control group and the ELb group (*p* < 0.01). The variations in concentrations, which provide insights into how the treatments impact pathways, are illustrated in Figure 3m.

### 3.3. Percentage Change in Dosed Parameter Effect (%) Compared with That in E, EThy and M Groups

The effect index measures the differences between the control group that had hyperthyroidism but did not receive any treatment, the absolute control group, and the group that was treated with thiamazole. This index allows for the evaluation of the efficacy of probiotic treatments, both individually and in combination. We determined the percentage change in hormone and neurotransmitter levels using the following formula:

(1) $$\mathrm{Effect}\;(\%)=(\frac{Value\;of\;experimental\;treatment-Value\;of\;control\;treatment}{Value\;of\;control\;treatment})\times 100$$

This formula allows for the assessment of parameter changes in the treatment groups relative to the control groups, as illustrated in Table 3. A positive value indicates an increase in the parameter compared with the control, while a negative value signifies a decrease.

It is worth mentioning that there was a rise in plasma cortisol levels following ELbSch treatment in contrast to group E, indicating a potential stress response or alteration in the regulation of the HPA axis. On the other hand, the EThy group exhibited an increase, whereas the ELb group demonstrated a decrease. The plasma levels of ACTH showed a significant increase in the ESch group compared with those in group E, suggesting a notable systemic response. Interestingly, although serotonin levels decreased in the preponderance of groups, the ESch group demonstrated an increase in the brain. Regarding brain norepinephrine levels, it was noted that the ELb group exhibited a higher increase compared with group E, indicating a potential boost in activity. However, the plasma samples revealed an increase in the ELbSch group. The levels of GABA in the brain remained relatively stable throughout the treatments, with minor fluctuations.

## 4. Discussion

Hyperthyroidism is a condition that implies low concentrations of thyrotropin (TSH, or thyroid-stimulating hormone) and the excessive production of triiodothyronine (T3) and/or free thyroxine (FT4), affecting 2.5% of adults worldwide [49]. Thyroid function is regulated by a negative feedback mechanism involving the hypothalamus and pituitary gland, together acting as a regulatory system, named the hypothalamus–pituitary–thyroid (HTP) axis [50]. It was concluded that thyroid hormones impact not only the HTP axis but other endocrine systems as well, particularly the HPA axis, laying the groundwork for intriguing interactions among various neuroendocrine systems [51]. Studies demonstrated a link between the HPT axis and the HPA axis, suggesting that thyroid hormones may have a role in controlling hypophyseal ACTH secretion and further influencing adrenal function [41,52]. Moreover, the complementary and inversely related effects of glucocorticoids on the HPT axis and of thyroid hormones on the HPA axis are acknowledged. Nevertheless, the mechanisms governing these interactions remain poorly understood, involving indirect influences on hypothalamic and pituitary modulation as well as hormonal metabolism and perhaps compensatory mechanisms [40].

Hyperthyroidism is associated with an increased risk of atrial fibrillation and osteoporosis. Treatment options for hyperthyroidism include one of the following approaches: antithyroid medications, therapy involving radioactive iodine, or thyroid surgery, depending on the severity of the case. Each one is adequate, but none is ideal, which is why novel methods to treat or support patients dealing with hyperthyroidism are emerging [53]. As probiotics are largely used by the human population due to their contribution to gut microbiota balance, digestive health improvement, and immune system support [54], they have been the subject of numerous studies trying to elucidate the full extent of probiotics’ therapeutic potential across various diseases and treatment modalities.

There is a limited amount of research concerning the correlation between probiotics, hyperthyroidism, and neurotransmitters as a whole. Huo et al. [55], for example, conducted research on the probiotic *Bifidobacterium longum*’s impact when supplied with methimazole, an antithyroid medication used to treat hyperthyroidism. Their study showed an improvement in the thyroid function of Graves’ disease patients due to the regulation of the gut microbiota and metabolites, which further impact neurotransmitter and blood trace elements through the gut–brain axis and gut–thyroid axis.

### 4.1. Evaluation of Neurohormonal Dynamics after Hyperthyroidism Induction

Hyperthyroidism (HT) is commonly linked with a range of changes in neurohormones, which reflect its effects on the endocrine and neurotransmitter systems [56]. Existing studies suggest that HT can cause modifications in the pituitary adrenal (HPA) and hypothalamic pituitary thyroid (HPT) axes, often leading to elevated levels of thyroid hormones in the circulation [57]. This, in turn, can impact neurotransmitter levels and hormonal balance throughout the body.

Inducing hyperthyroidism in our murine model led to significant alterations. Notably, we observed a 45% increase in ACTH levels within the brain, accompanied by an approximately 75% decrease in plasma ACTH concentrations. This contrast between ACTH levels in the brain and plasma might indicate the mechanisms of the HPA axis when exposed to excessive thyroid hormones. The decline in ACTH levels within the plasma observed in our study supports the effectiveness of the hyperthyroidism model. It suggests that HPA axis suppression can activate the HPT axis through mechanisms like increased thyroxine 5′ deiodinase type II enzyme activity [42]. This interaxis communication highlights how thyroid and adrenal regulation closely interact, with implications for both metabolic and psychological well-being.

Furthermore, serotonin (5HT) and dopamine (DA) levels experienced fourfold and threefold increases, respectively, within the brain, suggesting a significant influence of thyroid hormone dysregulation on mood and cognition-related neurotransmitter systems [58]. Thyroid hormones have the potential to affect dopamine receptor expression within the brain, potentially leading to changes in dopamine signalling dynamics. Increased receptor density or sensitivity could magnify the impact of dopamine, causing its functional effect to appear higher even if the actual levels are not significantly elevated [59,60].

Our results also showed that histamine (Hys) levels in the blood doubled. We can thus formulate an opinion on hyperthyroidism as a condition that increases the risk for allergic reactions. So far, only a few studies have linked allergies to autoimmune thyroid diseases, like Hashimoto’s thyroiditis and Graves’ disease, associated with high IgE levels [61,62].

Another interesting result is represented by gamma-aminobutyric acid (GABA), which increased by 40% in the brain and 80% in the blood plasma. Although GABA is an inhibitory neurotransmitter unlikely to be associated with hyperthyroidism, the positive variations in GABA levels in both the brain and plasma found in our study align with those found by Wiens et al., who discovered that thyroid hormones could stimulate GABA release, suppress GABA reuptake via extranuclear actions, and prolong GABA activity [63]. Additionally, plasma adrenaline (A) and norepinephrine (NA) levels were approximately three times higher, indicating an influence of hyperthyroidism on both peripheral and neurohormonal balance. Our finding supports the statement that high catecholamine levels represent a well-established feature of hyperthyroidism and are released due to excess thyroid hormones [64]. Furthermore, our findings provide evidence for a connection between thyroid function and mental health, as levels of 5HT and NA—commonly associated with anxiety disorders—were detected in large amounts in brain samples, in amounts four times higher than in healthy models for both neuromediators. After receiving thyroid treatment, the presence of changed levels of neurotransmitters can still be observed. This is evident in the rise in both 5HT and NA levels after thiamazole administration. It emphasises the lasting effects caused by thyroid dysregulation [65].

### 4.2. Effects of Lactobacillus acidophilus Administration in an Experimental Hyperthyroidism Model

The group treated with *Lactobacillus acidophilus* (ELb) showed a 50% increase in brain cortisol levels compared with the control group, followed by a 12% decrease in plasma and a 15% decrease in plasma cortisol when compared with the EThy group. These findings suggest that *Lactobacillus acidophilus* may have an impact on the response of the HPA axis to hyperthyroidism. This effect is likely due to the interaction between the probiotic and the gut–brain axis, which could potentially help normalise HPA axis overactivity by improving gut health and reducing inflammation [66].

Furthermore, the ELb group exhibited an increase in plasma ACTH levels compared with the E group. There was no significant change when compared with the EThy group. This suggests that *Lactobacillus acidophilus* has an influence on hyperthyroidism, which is comparable with the effect of thiamazole when it comes to ACTH levels. It appears that this probiotic can affect how the HPA axis is regulated, enhancing ACTH secretion and perhaps having a role in reinstating host euthyroid status. These findings also might indicate the targeted role of *Lactobacillus acidophilus* in regulating stress hormones, as variations in cortisol closely follow ACTH pulses [67]. However, plasma cortisol levels following the administration of the *Lactobacillus* strain did not vary much when compared with those in the E and Ethy models. The only relevant enhancement, which is also in line with the previous assumption, can be observed in brain cortisol levels, with a 50% increase compared with the baseline (E). 

Moreover, when administering *Lactobacillus* strains, there was a 50% decrease in 5HT levels in the brain compared with the baseline (E) and a slightly larger decrease of 59% compared with those in the EThy model. The observed decrease can be attributed to the interaction between the gut microbiota and the central nervous system, known as the gut–brain axis. This reduction may occur due to changes in tryptophan metabolism as *Lactobacillus* strains potentially increase the activity of enzymes that redirect tryptophan away from production and towards the kynurenine pathway [68,69]. Moreover, these probiotics produce short-chain fatty acids (SCFAs) [70,71], which indirectly affect neurotransmitter dynamics by influencing inflammation, the permeability of the blood–brain barrier, and neurotrophic factors such as brain-derived neurotrophic factors that impact pathways [72]. The modulation of responses by *Lactobacillus* strains might also influence serotonin levels by regulating profiles associated with enzymes involved in serotonin metabolism [73]. Furthermore, *Lactobacillus* directly produces compounds and alters neural signals from the gut to the brain through the vagus nerve [74,75], significantly impacting both synthesis and degradation.

The significant reduction in DA levels in the brain and plasma by 75% and 25%, respectively, compared with those levels in the control group, E, indicates that *Lactobacillus acidophilus* has an impact on neurotransmitter systems, including those involved in reward and motor functions. This could also affect the interaction between the HPA and HPT axes, potentially influencing stress response and thyroid function regulation. On the other hand, it is possible that the observed decrease in DA levels does not directly contribute to hyperthyroidism’s pathophysiology but serves as an adaptive reaction to altered neuroendocrine dynamics. The organism may reduce DA production in response to the increased activity of the thyroid and sympathetic nervous systems in order to maintain equilibrium [76].

Moreover, we observed an increase in histamine (Hys) plasma levels in the ELb group—tripling compared with that in the control group E and doubling relative to that in the EThy group. *Lactobacillus acidophilus* stimulates the release of histamine, an effect that can be realised by favouring allergic events; we can thus formulate an opinion regarding the risks of associating probiotics containing *Lactobacillus acidophilus* with potentially allergenic food products. The growth of histamine levels could be clinically relevant for patients who have a low expression of histamine-degrading enzymes like diaminoxidase (DAO) and histamine N-methyltransferase (HNMT) [77]. This potential allergenic effect of *L. acidophilus* is aligned with other studies that recognise *Lactobacillus* strains as being producers of this biogenic amine [78,79]. On the other hand, numerous studies confirm the ability of *Lactobacillus* bacteria to avoid allergic reactions through mechanisms like binding histamine, making these probiotics suitable candidates against histamine poisoning [80]. Additional research is required to draw a definitive conclusion regarding the involvement of *Lactobacillus* strains in allergic reactions. However, our study provides insight into the potential of *Lactobacillus acidophilus* to excessively stimulate the release of Hys, which may contribute to the onset of allergies in individuals with hyperthyroidism.

Our study observed a 50% decrease in adrenaline levels compared with those in the the hyperthyroidism group and a 33% increase compared with those in patients with hyperthyroidism who were treated with thiamazole. Probiotics might have an impact on adrenaline metabolism or indirectly influence adrenal function through the modulation of the HPA axis, which helps reduce adrenal stimulation, commonly associated with hyperthyroid conditions [81]. In different terms, *Lactobacillus acidophilus* proved its role in normalising the elevated stress response in hyperthyroidism. The increased levels of A when compared with that in the EThy model might show that the standard treatment is a better choice when it comes to the regulation of stress chemicals in this disease.

The increased levels of brain noradrenaline, counting 62% and 44%, respectively, are also notable, when compared with those in both the E and EThy models, effects that could signify the central positive modulation of this neuromediator by the *Lactobacillus* strain, independent of disease or its standard treatment. This is particularly interesting, as the HTP axis plays a fundamental role in mood regulation. Notably, there was an association between clinical depression and hyperthyroidism, which may be due to the chronic nature of this thyroid disease, which enhances the risk of the patient becoming depressed [82]. Major depressive disorder may also occur due to a dysfunctional central noradrenergic system manifested by the decreased synthesis of NA, the reduced density of its transporter in the locus coeruleus, and heightened tyrosine hydroxylase activity among individuals with depression [83]. We can thus form the conclusion that *Lactobacillus acidophilus* could benefit depression-suffering patients through this mechanism of central sympathetic system activation response.

However, plasma NA levels in the ELb group increase relative to those in the E model and decrease relative to those in the EThy model. As lower levels of NA have been associated with depression in hyperthyroidism patients, this may suggest that *Lactobacillus* does not manifest a better regulation of peripheral NA levels when compared with standard thiamazole treatment.

### 4.3. Effects of Saccharomyces boulardii Administration in an Experimental Hyperthyroidism Model

The administration of *Saccharomyces boulardii* showed that it can affect hormone levels, indicating its potential for regulating stress and neuroendocrine responses. Interestingly, the group that received *Saccharomyces boulardii* exhibited an increase in cortisol levels in the brain compared with the hyperthyroid group, suggesting that it may have an enhanced stress response or an adaptive mechanism to counteract the stress caused by hyperthyroidism. On the other hand, when comparing ESch with E, there was a decrease of 66% in plasma cortisol levels and a decrease of 67% when compared with those in the thiamazole-treated hyperthyroid group. These results indicate that *Saccharomyces boulardii* may have an effect on overall stress markers.

Furthermore, treatment with *Saccharomyces boulardii* led to an impressive increase in plasma ACTH levels, which were fivefold higher than those observed in group E and double those observed in group EThy. This notable increase in ACTH, along with decreased plasma cortisol levels compared with those in both the E and EThy models, may suggest a discontinuity of the negative feedback mechanism of the HPA axis and a compensatory mechanism, which is more visible in the yeast strain model than the previously disputed bacterial strain model. Similar to *Lactobacillus acidophilus*, but on a higher grade, increases in ACTH and cortisol concentrations induced by *Saccharomyces boulardii* demonstrate the activation of the HPA axis and subsequent inhibition of the HPT axis, which is a desired effect in hyperthyroidism.

The ESch group displayed a doubling of brain 5HT levels compared with those of the E group, suggesting that the serotoninergic system is activated following the administration of *Saccharomyces boulardii*. Other previous studies have provided support for the effect of serotoninergic up-regulation [84,85]. *Saccharomyces boulardii* sensitises enterochromaffin cells (ECs) with the consequence of excess serotonin release. 5-HT biosynthesis within the GI tract is an important factor that contributes to the overall serotonin present in the body, but the mechanisms that regulate the metabolism of gut-derived 5-HT are not completely known. It is well known that ECs are the largest producers of 5HT in the human body, accounting for more than 90% of the body’s serotonin. ECs can act as bidirectional transmitters between the gut and the brain through the contact of serotonin with specific primary afferent nerve fibres, playing a fundamental role in cognition, depression, and chronic pain. The other part of the 5-HT secreted by ECs can be taken by the blood circulation to the brain [86]. Whether some gut microorganisms may play a role in the de novo synthesis of 5-HT is also an aspect not elucidated. Still, the interactions that happen between the host and microbiota reveal growing evidence that the gut microbes could modulate gastrointestinal physiology by signalling to host cells. Some bacterial species such as *Escherichia coli*, *Streptococcus* spp., and *Corynebacterium* spp. were reported as being capable of 5-HT synthesis via the decarboxylation of gut tryptophan to tryptamine. Tryptophan hydroxylase is also a key enzyme in serotonin synthesis. The microbiota that possesses this enzyme activity is also supposed to be able to upregulate peripheral 5-HT [87]. Our study reveals *Saccharomyces boulardii* as a new microorganism capable of producing serotonin and stimulating its synthesis. We can thus form an opinion on the cautious administration of probiotics with *Saccharomyces boulardii* in patients with hyper-serotoninergic pathologies or who receive serotonergic agents (drugs or supplements) that can lead to serotoninergic syndrome.

In comparison, there was a decrease of 89% in brain DA levels compared with those in both the E and EThy groups. This suggests a reduction in signalling, which could have implications for motor activity and reward processing. The observed decline in DA levels may represent a response to maintaining balance, despite the changes caused by the effects of hyperthyroidism on the thyroid and sympathetic nervous systems.

The ESch group showed a significant decrease in Hys levels in plasma compared with those in the E group, with an even greater reduction of 60% compared with those in the EThy group. This decrease may indicate a reduction in inflammation or immune response modulation induced by probiotics [88]. Considering the role of histamine in hypersensitivity reactions and inflammation, this modulation could be beneficial. It is of particular interest that *Saccharomyces boulardii* successfully normalised Hys plasma levels, while *Lactobacillus acidophilus* did not show this effect. Our study highlights that administering *Saccharomyces boulardii* strains can help prevent events in patients with atopic terrain without significantly affecting histamine levels in plasma. Thus, allergic risk can be avoided or reduced by administering that *Saccharomyces boulardii* strain, which in our study generated histamine levels that were similar to those in the healthy model and twice lower than those in the HT model. This can mean that in cases of a raised allergic profile due to HT, probiotics containing *Saccharomyces boulardii* can be administered as a treatment variant for eventual allergic reactions. There are very few studies that state a relationship between allergy and HT-associated disorders [89,90]; thus, the allergic reactions that appeared in patients with an HT background have not been properly assessed so far.

The ESch group follows the same path as the ELb group, as the variations in NA levels are visible but less pronounced than those in the ESch group. NA levels increased by 40% in the brain of the Esch group compared with those in the E group and by 22% compared with those the EThy group. Additionally, there was a 20% increase in plasma levels when compared with the E group and a 20% decrease when compared with the EThy group. Therefore, both probiotics used in this study show the same influence on the sympathetic nervous system, particularly at the brain level, where the central modulation of neuromediators could be a real advantage for the management of mental diseases associated with hyperthyroidism.

Furthermore, it was found that plasma A levels in the ESch group were almost the same compared with those in the E model and 2.5 times higher compared with those in the EThy group. This shows that *Saccharomyces boulardii* has no beneficial effect on the modulation of A levels when added as prophylaxis or adjuvant therapy to hyperthyroid patients and also has no advantage in comparison with standard treatment for this disease.

### 4.4. Effects of Lactobacillus acidophilus and Saccharomyces boulardii Administration in an Experimental Hyperthyroidism Model

Upon comparing the group of animals that received the combination of probiotics to the hyperthyroidism group, a clear enhancement in stress response mechanisms was observed. Cortisol levels in the brain doubled, while in plasma, they tripled. These findings indicate the notable activation of the HPA axis, potentially attributed to the impact of probiotics on gut–brain communication.

The ACTH also experienced an increase, with brain ACTH levels rising by 50% and plasma ACTH levels tripling. These changes indicate an intensified response to stress at both peripheral and central levels, further supporting activity within the HPA axis and consecutively inhibiting the HPT axis, as the two microorganisms were observed to do when administered in separate forms.

The levels of neurotransmitters in the brain that are essential for mood regulation and cognitive function, including 5HT and DA, increased by approximately 30%. The administration of probiotics has a notable effect on the levels of neurotransmitters, which are typically regulated by the gut microbiota. This is consistent with other previous studies [91,92]. Nevertheless, the results obtained emphasise the importance of tailoring probiotic therapy based on specific pathologies and concurrent medications to account for potential interactions between probiotics and drugs. It can therefore be stated that hyperthyroidism is a pathology in which the administration of probiotic strain combinations should be carried out with caution in patients at risk of serotonin syndrome, with much greater safety being provided by microorganisms administered singly.

Plasma histamine levels experienced a significant increase of 90% when compared with those in E and a notable rise of 35% when compared with those in EThy. Thus, the administration of the two microbial strains had an additional effect, considering that our study previously demonstrated a high increase in *Saccharomyces boulardii* and a significant decrease *in Lactobacillus acidophilus* in Hys levels. This finding also indicates that the allergenic effect of *Lactobacillus* strains may be diminished via concomitant administration with the *Saccharomices* strain. Still, caution in probiotic combination administration for patients with atopic terrain is needed.

The treatment with *Lactobacillus acidophilus* and *Saccharomyces boulardii* caused changes in the adrenergic system, with a decrease in plasma A levels from 500 ng/mL to 212 ng/mL (a 50% reduction) and an increase in plasma NA levels from 1.63 ng/mL to 2.78 ng/mL (a 70% increase). These changes may indicate a readjustment of the response of the system to stress and metabolic regulation. Also, almost no variations were observed in A and central and peripheric NA levels compared with those in the EThy model, a fact that indicates that the probiotic duo may have the closest effect on the sympathetic system to that on the EThy group, indicating the comparable action of microorganisms on NA and A levels with the normal treatment of hyperthyroidism.

We can thus affirm that the probiotic microbial strains investigated contribute to the modulation of the HPA and HPT axes in hyperthyroid patients. Furthermore, our study highlights the importance of considering probiotic strains as a supplement to enhance overall thyroid function, as probiotic therapy may have the potential to positively impact thyroid hormones. Some precautions should be taken as our findings revealed a marked influence of probiotics over both central and peripheral neurotransmitters and hormones. The selection of bacteria or yeast to use as prophylaxis or adjuvant treatment for hyperthyroid patients should be carefully examined and specific to each patient, depending on their comorbidities and risk of developing any particular reactions.

**Figure 4:**
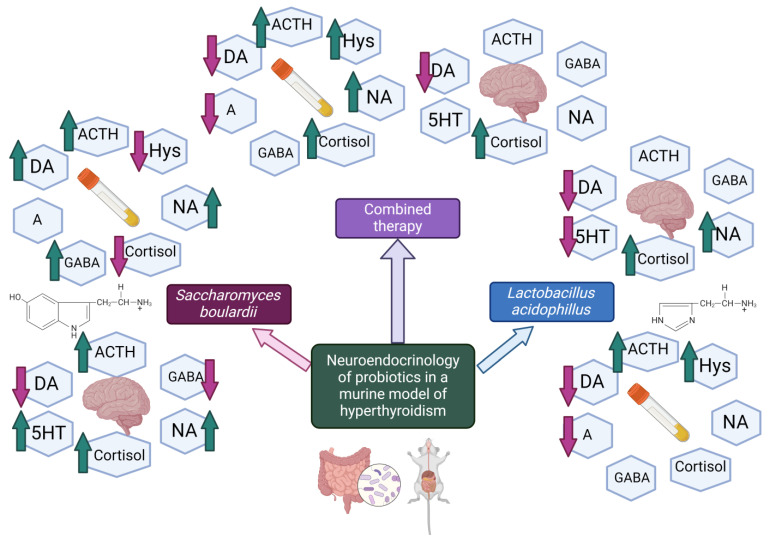
Neuroendocrinology of probiotics—brain and plasma modifications (created with Biorender.com, accessed on 20 March 2024). **Legend:** ↑: increase; ↓: decrease.

In summary, this study’s findings shed light on the potential therapeutic benefits of using strains such as *Lactobacillus acidophilus* and *Saccharomyces boulardii* in a hyperthyroid murine model. Specifically, the research offers valuable insights into the regulation of histamine and serotonin, opening up novel options for treatment. Figure 4 represents a graphical summary of the present study.

It was observed that *Lactobacillus acidophilus* can increase histamine levels, indicating that caution should be exercised when using it in individuals with conditions or those prone to allergies as it may worsen histamine-related reactions. This finding emphasises the need for consideration when incorporating probiotics into treatments for patients with allergic tendencies or when used alongside medications that react to histamine. On the other hand, *Saccharomyces boulardii* exhibited the ability to decrease histamine levels, suggesting its potential usefulness in alleviating allergic responses and potentially serving as a complementary therapeutic agent for managing atopic disorders or reducing side effects caused by interactions with histamine-related drugs.

In addition, this study has provided valuable insights into the effects of *Saccharomyces boulardii* on serotonin dynamics, which may result in higher levels of 5HT compared with those of other probiotic treatments and controls.

**Figure 5:**
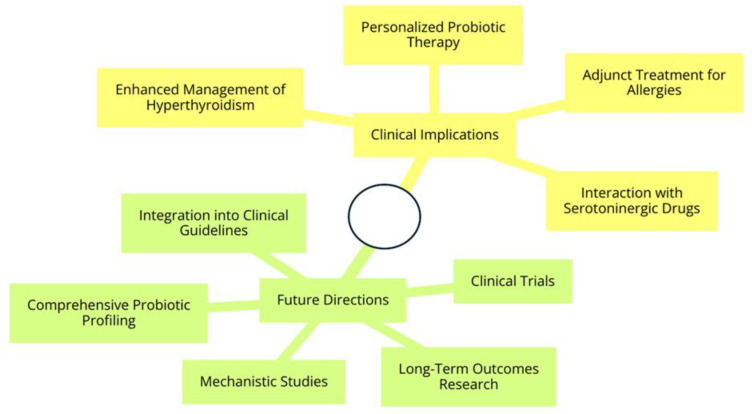
Clinical implications and future directions.

In general, these findings enhance our understanding of how probiotics affect dynamics, which has applications for developing safer and more efficient treatment approaches. This is particularly relevant for conditions involving the endocrine system and immune responses. Figure 5 illustrates the clinical implications and future directions of our study. This supports the idea of probiotic supplementation alongside pharmacotherapy.

## 5. Conclusions

In our study, the assessment of neurotransmitter levels provides a comprehensive view of the neurotransmitter environment in a murine model, offering insights into the complex interplay between neurotransmitter balance, probiotics, and hyperthyroidism. By analysing oscillations in dopamine, GABA, serotonin, histamine, noradrenaline, and adrenaline, we targeted the main neurochemicals that may play a role in the gut microbiota–thyroid connection. This way, we assessed to what extent probiotic administration can constitute an impact factor in prophylaxis and also in modulating already established hyperthyroid disease. This study not only expands our knowledge and further understanding of the intriguing relationship between hyperthyroidism and specific neurotransmitters but also sheds light on future probiotic therapeutic approaches and the vigilance that may be required.

## Figures and Tables

**Table 1:** Scoring system for the assessment of animal conditions.

Score	Parameters
Fur	
[0]	Clean and smooth; no signs of neglect or damage.
[1]	Slightly unkempt; may have some areas that are more matted.
[2]	Matted or with missing portions; visible signs of neglect.
[3]	Visible lesions, extensive areas without fur, signs of self-mutilation.
Eyes	
[0]	Clear and open; no discharge or redness.
[1]	Slight discharge or slight redness.
[2]	Obvious discharge or redness; signs of irritation.
[3]	Closed, inflamed, or showing signs of infection or injury.
Activity	
[0]	Normal, active, moving freely.
[1]	Slightly lethargic; some changes in mobility.
[2]	Lethargic, with significant difficulty moving.
[3]	Immobility; unable or unwilling to move.
Food and water consumption	
[0]	Normal, consumes usual amounts of food and water.
[1]	Slightly reduced; we observe a decrease in interest in food and water.
[2]	Significantly reduced; consumes very little food and water.
[3]	Complete refusal; does not consume food or water.
Aggressiveness	
[0]	Calm, normal behaviour: the animal interacts peacefully; there are no signs of unwarranted aggressive or defensive behaviour.
[1]	Easily irritable: shows minor signs of irritability when provoked or under stress. These may include avoiding contact or sudden movements, but without resorting to aggression.
[2]	Moderately aggressive: shows sporadic aggressive behaviours such as biting, scratching, or pushing other animals, especially in situations of competition for resources or space.
[3]	Highly aggressive: frequently exhibits aggressive behaviours, even in the absence of direct provocation. It can be dangerous for congeners or for people who take care of the animals. Aggressiveness is evident even without the pressure of external stressors.
Interpretation of the final score	
Score 0–0.5	Excellent state of health, without signs of discomfort or stress.
Score 0.6–1.5	Slight concern; needs further monitoring.
Score 1.6–2.5	Moderate health problems may require interventions.
Score 2.6–3.0	Severe health problems, requiring immediate intervention.

**Table 2:** Mean concentration values of dosed markers in brain and plasma samples from experimental groups of the small rodent hyperthyroidism model (N/A—data not available).

Mean ± SD	ELb	ESch	ELbSch	E	EThy	M
Cortisol Brain HT (ng/mL)	1.63 ± 1.56	3.18 ± 0.90	2.21 ± 1.12	1.08 ± 0.93	N/A	N/A
Cortisol Plasma HT (ng/mL)	19.03 ± 10.31	7.29 ± 5.42	64.2 ± 33.68	21.49 ± 6.65	22.47 ± 18.11	N/A
ACTH Brain HT (pg/mL)	1.35 ± 0.68	1.54 ± 1.26	2.1 ± 1.12	1.51 ± 0.91	1.43 ± 0.95	1.05 ± 0.79
ACTH Plasma HT (pg/mL)	28.27 ± 2.96	53.18 ± 17.06	33.13 ± 2.98	11.55 ± 5.84	26.03 ± 1.96	45.14 ± 5.03
Serotonin Brain HT (ng/mL)	0.77 ± 0.23	3.21 ± 0.19	2.03 ± 1.09	1.55 ± 2.04	1.88 ± 1.45	0.41 ± 0.61
Dopamine Brain HT (pg/mL)	10.35 ± 6.69	4.54 ± 0.92	52.66 ± 35.20	41.23 ± 1.33	43.19 ± 1.89	14.18 ± 6.41
Dopamine Plasma HT (pg/mL)	1228 ± 102.5	2140 ± 357.5	1103 ± 180.8	1743 ± 142.6	1578 ± 161.8	N/A
Hys Plasma HT (ng/mL)	4.11 ± 2.24	0.73	2.76	1.44 ± 0.20	2.02 ± 0.21	0.73 ± 0.80
GABA Brain HT (pg/mL)	307.8 ± 13.65	293.8 ± 37.31	314.8 ± 88.55	311.8 ± 15.48	280.6 ± 32.38	228.4 ± 85.84
GABA Plasma HT (pg/mL)	3106 ± 873.3	3569 ± 978.2	3363 ± 507.9	3287 ± 590.2	4522 ± 2477	1819 ± 676.6
A Plasma HT (ng/mL)	254.4 ± 68.35	504.4 ± 88.93	212.8 ± 26.59	499.8 ± 62.37	190.2 ± 98.74	183.7 ± 82.70
NA Brain HT (ng/mL)	0.13 ± 0.01	0.11 ± 0.02	0.10 ± 0.05	0.08 ± 0.05	0.09	0.02 ± 0.01
NA Plasma HT (ng/mL)	1.94 ± 0.71	2.08 ± 0.17	2.78 ± 0.15	1.63 ± 0.73	2.61 ± 0.35	0.59

Legend: ACTH: adrenocorticotropic hormone; Hys: histamine; GABA: gamma-aminobutyric acid; A: adrenaline; NA: noradrenaline; HT: hyperthyroidism; ELb: experimental group receiving Lactobacillus acidophilus; Esch: experimental group receiving Saccharomyces boulardii; ELbSch: experimental group receiving combined probiotic therapy; E: hyperthyroidism control; EThy: hyperthyroidism group treated with thiamazole; M: absolute control.

**Table 3:** Percentage variation in the dosed parameters’ % effect compared with that in groups E, EThy, and M.

Effect (%) vs. E	Elb vs. E (%)	ESch vs. E (%)	ELbSch vs. E (%)	EThy vs. E (%)	M vs. E (%)
Cortisol Brain HT (ng/mL)	50.93	194.44	104.63	N/A	N/A
Cortisol Plasma HT (ng/mL)	−11.45	−66.08	198.74	4.56	N/A
ACTH Brain HT (pg/mL)	−10.60	1.99	39.07	−5.30	−30.46
ACTH Plasma HT (pg/mL)	144.76	360.43	186.84	125.37	290.82
Serotonin Brain HT (ng/mL)	−50.32	107.10	30.97	21.29	−73.55
Dopamine Brain HT (pg/mL)	−74.90	−88.99	27.72	4.75	−65.61
Dopamine Plasma HT (pg/mL)	−29.55	22.78	−36.72	−9.47	N/A
Hys Plasma HT (ng/mL)	185.42	−49.31	91.67	40.28	−49.31
GABA Brain HT (pg/mL)	−1.28	−5.77	0.96	−10.01	−26.75
GABA Plasma HT (pg/mL)	−5.51	8.58	2.31	37.57	−44.66
A Plasma HT (ng/mL)	−49.10	0.92	−57.42	−61.94	−63.25
NA Brain HT (ng/mL)	62.50	37.50	25.00	12.50	−75.00
NA Plasma HT (ng/mL)	19.02	27.61	70.55	60.12	−63.80
**Effect (%) vs. EThy**	**ELb vs. EThy (%)**	**ESch vs. EThy (%)**	**ELbSch vs. EThy (%)**	**E vs. EThy (%)**	**M vs. EThy (%)**
Cortisol Brain HT (ng/mL)	N/A	N/A	N/A	N/A	N/A
Cortisol Plasma HT (ng/mL)	−15.31	−67.56	185.71	−4.36	N/A
ACTH Brain HT (pg/mL)	−5.59	7.69	46.85	5.59	−26.57
ACTH Plasma HT (pg/mL)	8.61	104.30	27.28	−55.63	73.42
Serotonin Brain HT (ng/mL)	−59.04	70.74	7.98	−17.55	−78.19
Dopamine Brain HT (pg/mL)	−76.04	−89.49	21.93	−4.54	−67.17
Dopamine Plasma HT (pg/mL)	−22.18	35.61	−30.10	10.46	N/A
Hys Plasma HT (ng/mL)	103.47	−63.86	36.63	−28.71	−63.86
GABA Brain HT (pg/mL)	9.69	4.70	12.19	11.12	−18.60
GABA Plasma HT (pg/mL)	−31.31	−21.07	−25.63	−27.31	−59.77
A Plasma HT (ng/mL)	33.75	165.19	11.88	162.78	−3.42
NA Brain HT (ng/mL)	44.44	22.22	11.11	−11.11	−77.78
NA Plasma HT (ng/mL)	−25.67	−20.31	6.51	−37.55	−77.39
**Effect (%) vs. M**	**E vs. M (%)**				
Cortisol Brain HT (ng/mL)	N/A				
Cortisol Plasma HT (ng/mL)	N/A				
ACTH Brain HT (pg/mL)	43.81				
ACTH Plasma HT (pg/mL)	−74.41				
Serotonin Brain HT (ng/mL)	278.05				
Dopamine Brain HT (pg/mL)	190.76				
Dopamine Plasma HT (pg/mL)	N/A				
Hys Plasma HT (ng/mL)	97.26				
GABA Brain HT (pg/mL)	36.51				
GABA Plasma HT (pg/mL)	80.70				
A Plasma HT (ng/mL)	172.07				
NA Brain HT (ng/mL)	300.00				
NA Plasma HT (ng/mL)	176.27				

## Data Availability

Data are contained within the article.
